# Novel Melt-Spun Polymer-Optical Poly(methyl methacrylate) Fibers Studied by Small-Angle X-ray Scattering

**DOI:** 10.3390/polym9020060

**Published:** 2017-02-13

**Authors:** Markus Beckers, Thomas Vad, Benjamin Mohr, Benjamin Weise, Wilhelm Steinmann, Thomas Gries, Gunnar Seide, Emmanuel Kentzinger, Christian-Alexander Bunge

**Affiliations:** 1 Institut für Textiltechnik, RWTH Aachen University, Otto-Blumenthal-Straße 1, 52074 Aachen, Germany; thomas.vad@ita.rwth-aachen.de (T.V.); benjamin.mohr@ita.rwth-aachen.de (B.M.); benjamin.weise@ita.rwth-aachen.de (B.W.); wilhelm.steinmann@ita.rwth-aachen.de (W.S.); thomas.gries@ita.rwth-aachen.de (T.G.); 2 Faculty of Humanities and Sciences—Biobased Materials, Maastricht University, Urmonderbaan 22, 6167 RD Geleen, The Netherlands; gunnar.seide@maastrichtuniversity.nl; 3 Jülich Centre for Neutron Science (JCNS-2), and Peter Grünberg Institute (PGI-4: Scattering Methods), Forschungszentrum Jülich, D-52425 Jülich, Germany; e.kentzinger@fz-juelich.de; 4 Hochschule für Telekommunikation Leipzig (HfTL), Gustav-Freytag-Str. 43-45, 04277 Leipzig, Germany; bunge@hft-leipzig.de

**Keywords:** material characterization, polymer optical fiber, graded-index profile, fiber fabrication, nanostructure, measurement technique, scattering, melt-spinning

## Abstract

The structural properties of novel melt-spun polymer optical fibers (POFs) are investigated by small-angle X-ray scattering. The amorphous PMMA POFs were subjected to a rapid cooling in a water quench right after extrusion in order to obtain a radial refractive index profile. Four fiber samples were investigated with small-angle X-ray scattering (SAXS). The resulting distance-distribution functions obtained from the respective equatorial and meridional SAXS data exhibit a real-space correlation peak indicative of periodic cross-sectional and axial variations in the scattering density contrast. Simple model calculations demonstrate how the structural information contained particularly in the equatorial distance distribution function can be interpreted. The respective results are qualitatively verified for one of the fiber samples by comparison of the model curve with the measured SAXS data. Eventually, the study confirms that the cross-sectional variation of the (scattering-) density is the main reason for the formation of radial refractive-index profiles in the POFs.

## 1. Introduction

Polymer optical fibers (POF), mostly made from poly-methyl methacrylate (PMMA), provide a robust and cost-effective alternative to glass fibers for short-reach data communication, but also in illumination and sensing applications [1]. Their main advantage is the relatively large diameter of up to several millimeters, which makes handling much easier than with tiny glass fibers of only several tens of micrometers, but requires different, continuous fabrication techniques. Depending on the kind of application, POFs should show different properties, such as low attenuation, controlled scattering, or a high bandwidth for data communication. Especially for the latter, graded-index (GI) fibers have been developed that feature decaying refractive-index profiles towards the outer regions of the fiber [2,3,4]. This requires special fabrication methods that most often involve a discontinuous preform process, by which a large version of the fiber with the correct refractive-index distribution is manufactured first and then drawn to a fiber in a second step [5].

**Figure 1:**
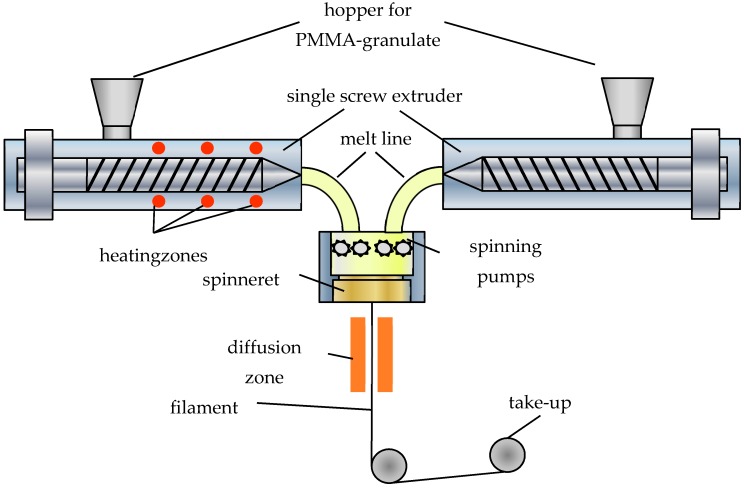
Example of a continuous POF fabrication process based on extrusion [5]. With this approach, only step-index fibers can be produced.

In [6], we presented a novel, continuous fabrication process, where the graded-index fiber is produced by a simple melt-spinning process without the use of any dopants to control the refractive index. The refractive-index profile is obtained by a subsequent rapid cooling of the filament right behind the spinning nozzle (see Figure 1). The different cooling speeds of the inner and outer regions lead to a radial density distribution, which results in a refractive-index profile without the use of any doping materials.

We could show already in [7] that the novel fabrication process without any doping materials can still lead to graded-index fibers with a distinct refractive-index profile. There are several established methods for the characterization of the refractive-index profile of fibers beginning with quantitative phase microscopy over the transmitted to the refracted near-field methods (RNF) to name just a few [8]. However, while the formation of a refractive-index profile by pure temperature treatment after the melt-spinning process could be proven, it has not been possible to provide evidence that this profile arises due to a density gradient and how the density is distributed within the fiber.

Surprisingly, the application of these methods to PMMA POFs produced by the aforementioned melt-spinning process determine the refractive-index profiles typical for GI-POFs although the POFs do not contain any dopants such as, e.g., bromobenzene, benzyl butyl, benzyl benzoate, phenyl sulfide, monomer additions, nanoparticles, such as TiO_2_ or fluorine, that are usually added to increase or decrease the refractive index over the cross-section of a POF according to their concentration levels [9,10,11,12]. The only reasonable explanation for the presence of a refractive index profile in a neat PMMA POF is the formation of a radial density gradient during the melt-spinning process, since both quantities are related to each other as described by the Clausius-Mossotti relation [13]:

(1) $$\rho \left(r\right)=\frac{M}{\rho_{\mathrm{mol}}}\frac{n^{2}\left(r\right)-1}{n^{2}\left(r\right)+2},$$

where *ρ* is the density, *M* = 100.12 g/mol is the molar mass of PMMA, *ρ*_mol_ = 24.751 × 10^−6^ m^3^/mol is the molar refractivity, *n* is the refractive index, and *r* denotes a possible dependence of the respective quantities on the radial distance *R* from the center of the fiber cross-section.

So far, a genuine proof of radial density gradients in the neat PMMA POFs is lacking. In order to obtain information on the origin of the measured refractive-index profiles, small-angle X-ray scattering (SAXS) was chosen, since this technique is ideally suited to detect density fluctuations on the nanoscale in the investigated material [14]. Since PMMA of optical grade is basically an amorphous polymer, SAXS experiments on neat PMMA POFs have not yet been performed (mainly) due to the fact that an amorphous material normally does not exhibit any small-angle scattering. Therefore, the present work is to be understood as an initial, explorative study to assess the possibility of detecting and verifying material density profiles over the fiber diameter via SAXS experiments.

The article is structured as follows: firstly, the materials and the novel fabrication method are described in detail in order to draw conclusions on the structural properties of the resulting fibers. Subsequently, the SAXS method is introduced and experimental conditions, as well as data-analysis techniques, are explained. The results are discussed, and the fundamental assumptions for the analysis of the SAXS curves are adapted in order to explain the structure parameters obtained from the different analysis methods. In summary, the results indicate the presence of cross-sectional density variations and, consequently, the general applicability of the SAXS method for the characterization of refractive index/density profiles in POFs.

## 2. Melt-Spinning Fabrication Process with Subsequent Cooling

*Polymer and polymer fiber processing*: Polymer optical fibers can be fabricated either by continuous or discontinuous processes, while continuous processes are the norm due to their low cost [15]. Usual continuous methods are extrusion, photo polymerization, and melt spinning. The first two processes generate the polymer during the fiber extrusion by polymerization that relies either on heat or light. The melt-spinning process, in contrast, uses already-fabricated polymer that is molten in order spin a fiber from that material (Figure 1). 

Since all process steps can be performed simultaneously it is possible to continuously generate a fiber of arbitrary length, i.e., the fiber can be produced without stops and therefore at extremely low cost. These processes are, however, not suitable for GI-POFs since each nozzle extrudes, more or less, homogeneous material of a specific refractive index without any profile. Therefore, one has to introduce a radius-dependent inhomogeneity.

Most processes for graded-index fibers are discontinuous preform techniques, in which the preform of the fiber is produced first and then drawn to a fiber in a second step. Since these steps have to be performed sequentially, only a fiber of limited length can be drawn from the preform, which makes this method more expensive.

**Figure 2:**
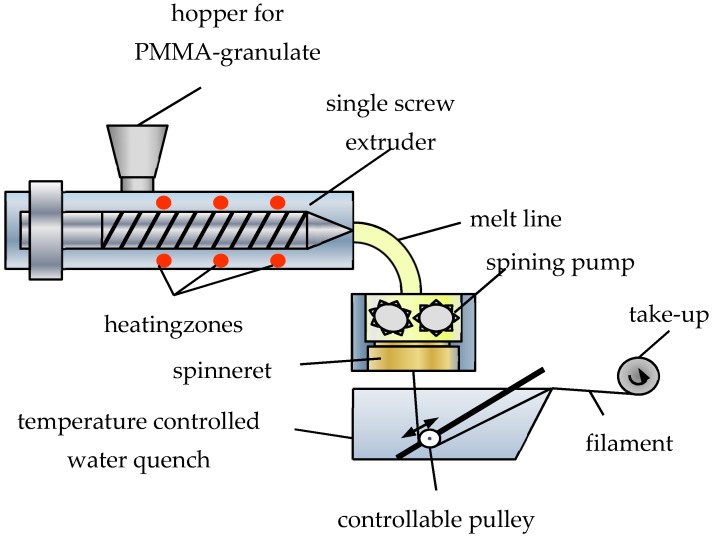
Schematic of the melt-spinning process based with subsequent rapid cooling in a water quench [16]. The still hot polymer filament is subjected to a rapid cooling in a water quench right below the spinning nozzle. The rapid cooling results in an inhomogeneous cooling speed over the fiber cross-section.

To achieve the graded-index profile in a continuous process, we used the melt-spinning technique with a subsequent rapid cooling in a water quench, which is supposed to introduce a radius-dependent cooling speed [16]. Figure 2 explains the process in detail. Using this continuous fabrication method, the fiber length is not limited. The main difference to the standard melt spinning is the water quench just after the spinning when the fiber is still warm and close to the glass-transition temperature.

With control of the cooling in the water quench, the refractive-index profile of the produced fiber can be engineered in a simple way using standard off-the-shelf production methods. The decrease of cooling speeds from the outer regions towards the fiber axis results in a density gradient with an increase of the density for lower cooling speed in the center of the fiber. This increasing density towards the fiber axis leads to a refractive-index profile according to the Clausius-Mosotti relation because of the larger number of polarizable material per volume. This density-gradient formation within the fiber, however, has not directly been proven. For this reason and because variations of the density can also lead to increased scattering [17], a study of the structural properties of these novel fibers have been conducted.

For the following study, fibers are spun from the amorphous optical grade PMMA PLEXIGLAS^®^ (Evonik Performance Materials GmbH, Darmstadt, Germany), Pure Optical Quality (POQ) with different process parameters, such as nozzle diameter, water and process temperature, and winding and extrusion speed. 

## 3. Fiber Analysis via Small-Angle X-ray Scattering Experiments

From an (ideal) amorphous substance, an a priori SAXS signal cannot be expected. Since semi-crystalline PMMA—which can be produced via Grignard reactions [18,19]—is not used for the production of POFs in order to avoid unwanted scattering effects at the crystalline/amorphous interface, only variations in the density of the material related to the refractive index profile (Equation (1)), and/or the presence of nanosized gas-inclusions and voids [20] can give rise to a SAXS signal. Hence, a clearly detectable small-angle scattering contribution from the (amorphous) POF is the crucial point for the applicability of the SAXS method to extract information on the refractive index profile from the measured data. 

Four POF samples produced with different sets of process parameters were selected for the SAXS experiments. Single processing conditions were not systematically varied for this initial study, and, therefore, only the complete production processes of the POF samples investigated by SAXS are different (see Table 1), which should nevertheless result in SAXS curves containing different (structural) information.

The spin-draw ratio λ_SD_ is the ratio between winding speed *v*_w_ and extrusion velocity *v*_e_. This value basically characterizes the mechanical forces which influence the PMMA nanostructure during the non-isothermal melt-spinning process, whereas the draw ratio λ_D_ corresponds to the length ratio between the stretched fiber *l*_D_ and as-spun fiber *l*_0_ achieved during an isothermal off-line drawing process performed at a constant temperature *T*_D_ above the glass-transition temperature of the polymer. 

**Figure 3:**
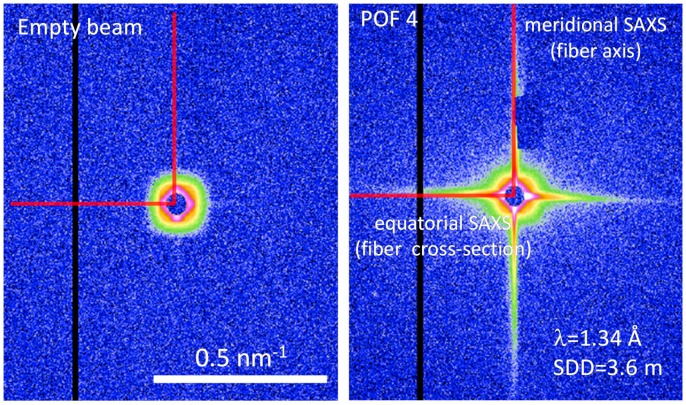
(**Left**) 2D-SAXS intensity distribution of the empty beam displaying an isotropic scattering. (**Right**) 2D-SAXS intensity distribution of the sample POF 4. The strong anisotropic equatorial and meridional scattering indicates that at least parts of the PMMA chains are highly oriented. Note that the color codes of empty beam and sample scattering are not to scale. The empty beam scattering intensity is on average about two orders of magnitude smaller than the small-angle scattering arising from the fiber sample.

The SAXS experiments were performed at the high brilliance laboratory Gallium Anode Low Angle X-ray Instrument (GALAXI) at the Jülich research center (Jülich, Germany) equipped with a BRUKER AXS MetalJet X-ray source (Madison, WI, USA) (Figure 3). The diffractometer is based on the former JUSIFA instrument installed at the DORIS-III storage ring at HASYLAB (Deutsches Elektronen Synchotron DESY, Hamburg, Germany) [21,22]. The data were recorded with a 1 M Pilatus detector (DECTRIS AG, Baden, Switzerland) at a sample-to-detector distance of 3.6 m and a wavelength of 0.134 nm covering a total range of momentum transfer of 0.03 nm^−1^ ≤ *q* ≤ 1.0 nm^−1^ (*q* = 4*π*sin(θ)/λ is the modulus of the scattering vector, 2θ is the scattering angle, and λ is the wavelength). Prior to data analysis, the collected data were reduced by normalization to the intensity of the transmitted beam and empty beam subtraction.

## 4. Results and Discussion

**Figure 4:**
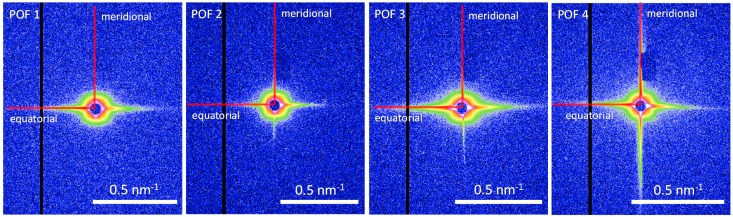
2D SAXS intensity distributions of the four PMMA POF samples. The anisotropic equatorial and meridional scattering is present in all samples, but clearly different, which indicates a high sensitivity of the fiber nanostructure to changes in the production processes.

**Figure 5:**
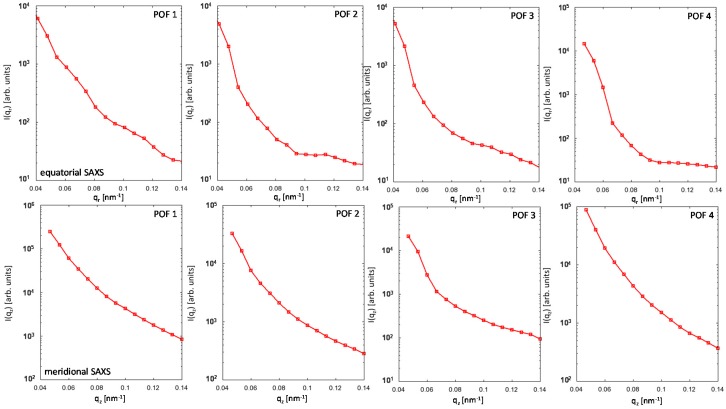
Extracted 1D SAXS intensity curves of the four POF samples. (**Top row**): equatorial intensities corresponding to the scattering from the fiber cross-section. (**Bottom row***)*: meridional intensities corresponding to the scattering contribution along the fiber axis.

For the four selected POF samples, a significant SAXS signal is detectable (see Figure 4) which basically confirms the presence of density variations in the amorphous PMMA fibers. Moreover, the significant differences in the 2D SAXS intensity distributions of the fibers demonstrate the influence of the processing conditions on the PMMA fiber nanostructure. The intensity streaks in the equatorial plane (corresponding to the fiber cross-section) and the meridional direction (corresponding to the fiber axis) appear to be quite sharp and indicate a high degree of axial orientation of the observed nanostructure. Since the major changes in the scattering intensities also take place perpendicular and parallel to the fiber axis, the respective equatorial and meridional intensity contributions were extracted (Figure 5) in order to facilitate the analysis by methods that are usually applied to 1D scattering curves—the Guinier approach and the distance distribution function [14]. Both methods have the advantage, that a particular nanostructure model is not required for the analysis of the SAXS data.

From the Guinier analysis, the forward scattering intensity *I*_0_ and the radius of gyration *R*_g_ are obtained by fitting a model function:

(2) $$I_{\mathrm{obs}}\left(q\right)=I_{0}\exp\left[-\frac{q^{2}R_{g}^{2}}{3}\right]$$

to the measured low-*q* scattering data *I*_obs_(*q*). Since the fiber scattering exhibits cylindrical geometry [23], possible density fluctuations across the fiber diameter may be of cylindrical shape as well, and, therefore, the radius of gyration resulting from the Guinier fit of the equatorial scattering curve can be interpreted as a cylinder radius *R*_c_ which is related to *R*_g_ by:

(3) $$R_{g}^{2}=\frac{R_{c}^{2}}{2}.$$

Correspondingly, the Guinier analysis of the meridional scattering curve yields a cylinder length *L*_c_ where:

(4) $$R_{g}^{2}=\frac{1}{12}L_{c}^{2}.$$

The obtained values can be cross-checked by computation of the Fourier transform of the scattering intensities, i.e., the equatorial and meridional 1D Patterson (distance distribution) function

(5) $$\gamma_{\mathrm{equ}}\left(r\right)=\frac{1}{Q_{\mathrm{equ}}}\int_{0}^{\infty }I_{\mathrm{equ}}^{\mathrm{obs}}\left(q_{r}\right)J_{0}\left(q_{r}r\right)q_{r}M\left(q_{r}\right)dq_{r}$$

and

(6) $$\gamma_{\mathrm{mer}}\left(r\right)=\frac{1}{Q_{\mathrm{mer}}}\int_{0}^{\infty }I_{\mathrm{mer}}^{\mathrm{obs}}\left(q_{z}\right)\cos\left(q_{z}r\right)M\left(q_{z}\right)dq_{z}$$

with

(7) $$Q_{\mathrm{mer}/\mathrm{equ}}=\int_{0}^{\infty }I_{\mathrm{mer}/\mathrm{equ}}^{\mathrm{obs}}\left(q_{z/r}\right)M\left(q_{z/r}\right)dq_{z/r},$$

where *q*_r_ and *q*_z_ are the momentum transfer vector components in the equatorial plane (fiber cross-section) and along the fiber axis, respectively, and *J*_0_ is the zero-order Bessel function of the first kind. In order to suppress series termination ripples arising from the limited *q*-range, a damping function *M*(*q*_z/r_) = exp[−*B*^2^*q*^2^_z/r_] is used [24,25]. The constant *B* is chosen such, that *M*(*q*_z/r_) = 0.05 at *q*_z/r_ = 0.2 nm^−1^. The analysis of the SAXS curves was carried out with self-written Fortran 90 programs. For the Guinier fits, the Levenberg-Marquardt routines MRQMIN and MRQCOF were employed [26].

For an ideal single-particle scattering in a two-phase system, the respective maximum dimensions are obtained at the first intersection of the Patterson functions with the abscissa, i.e.:

(8) $$\gamma_{\mathrm{mer}/\mathrm{equ}}\left(r=D_{\mathrm{mer}/\mathrm{equ}}^{\mathrm{MAX}}\right)=0.$$

For the more general case, the maximum dimension is determined by the intersection of the linear extrapolation of γ(*r*) close to the abscissa and its first minimum [27,28].

**Figure 6:**
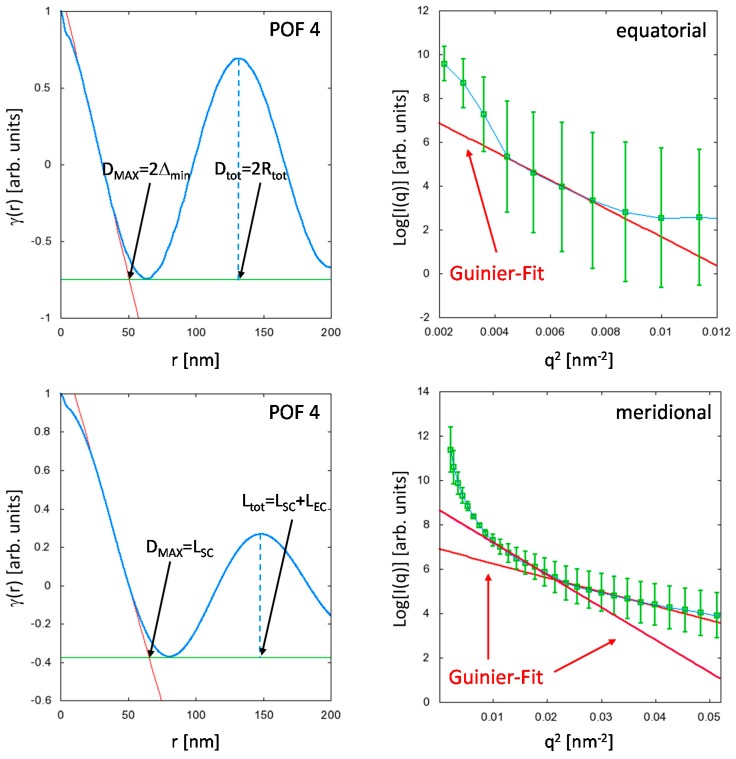
Analysis of the equatorial (**Top row**), and meridional (**Bottom row**) scattering contributions using the Guinier fit and the Patterson function exemplarily shown for the sample POF 4. Note that *r* corresponds to a distance, i.e., for the analysis of the equatorial SAXS intensities, *D*_MAX_ = 2Δ_min_, and for the meridional SAXS, *D*_MAX_ = *L*_SC_. The correlation peaks in the meridional and equatorial Patterson functions correspond to periodically occurring density variations along the fiber axis and the fiber cross-section, respectively.

The results are exemplarily shown for the sample POF4 in Figure 6. A striking feature is the similarity between the meridional and the equatorial Patterson functions. Both functions exhibit a real-space correlation peak which is related to a periodically occurring repeat unit. The presence of these correlation peaks is a quite unusual phenomenon for a generally amorphous material. Consequently, the peaks are very likely caused by periodic (scattering-) density variations along the fiber axis, as well as over the fiber cross-section. 

The meridional case is well known and has been extensively studied for the case of semi-crystalline polymer fibers [29,30]. Here, the maximum dimension *D*_MAX_ = *L*_cryst_ corresponds to the axial dimension of the polymer crystallite, while the peak in the meridional Patterson function can be assigned to the so-called long-period *L*_tot_ = *L*_cryst_ + *L*_amorph_, i.e., two adjacent crystallites are separated by amorphous polymer chain segments of length *L*_amorph_.

Although flow- or shear-induced crystallization effects can be excluded for the PMMA fibers, melt-spinning, however, produces straight polymer chain sections of length *L*_SC_, which—in comparison to the entangled polymer chain sections of length *L*_EC_ that separate two consecutive straight polymer sections—differ in their axial scattering densities, i.e., *L*_tot_ = *L*_SC_ + *L*_EC_. These straight chain sections can be considered as amorphous shish-precursors, which finally lead for a semi-crystalline polymer to the formation of polymer crystallites [31,32,33].

The sum of the lengths (*L*_SC_ + *L*_EC_) obtained from the analysis of the two different Guinier regions (Figure 6) agree quite well with the positions *L*_tot_ of the correlation peaks in the meridional Patterson functions for all POF samples and demonstrate that both Guinier and real space analysis yield consistent results (see Table 2). 

**Figure 7:**
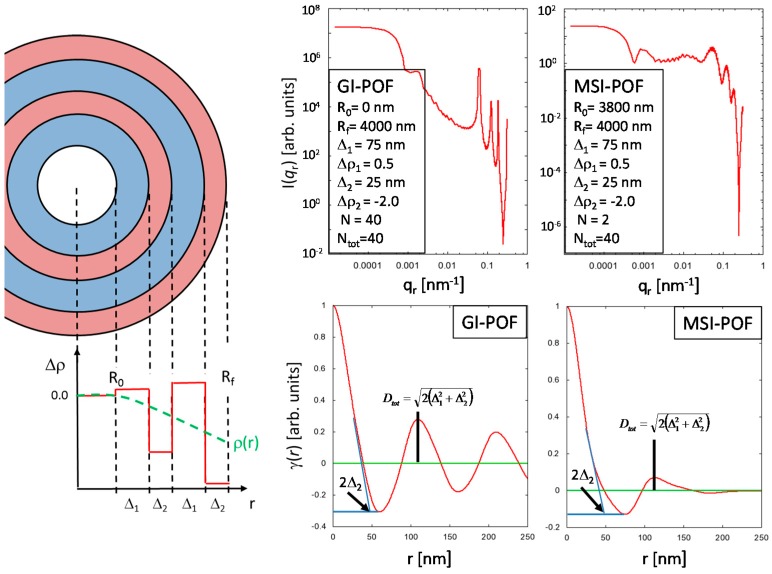
(**Left**) simple structure model for the description of radially decaying periodical density variations over the fiber cross-section. (**Top right**) equatorial SAXS intensity patterns of a model GI-POF and a two-step MSI-POF calculated with the structure model sketched on the left. The respective model parameters are displayed in the insets. For better visibility, high frequency oscillations in the SAXS curves were suppressed by a seven-point moving average. (**Bottom right**) equatorial distance-distribution functions for GI- and MSI-POF. The information content is essentially the same. In both cases, the smaller dimension Δ_2_ and the parameter *D*_tot_ can be extracted from the functions.

The interpretation of the equatorial Patterson function is less straightforward. Similar to the meridional distance-distribution function, the equatorial real-space correlation peak indicates the presence of a radially symmetric repeat unit, which can, e.g., be defined as two concentric ring-segments of widths Δ_1_ and Δ_2_ that exhibit significantly different scattering density contrasts Δ*ρ*_1_, and Δ*ρ*_2_, respectively. Moreover, good axial transmission properties of the POFs require that the refractive index and, according to the Clausius-Mossotti Equation (1), the material density, decays with increasing distance *r* from the fiber axis, which defines the general properties of the ring segments Δ_1_ and Δ_2_. The ring-segment of larger width (Δ_1_) can be assigned a positive scattering density contrast Δ*ρ*_1_ while the ring-segment with smaller width (Δ_2_) exhibits a negative scattering density contrast Δ*ρ*_2_ < 0. Thus, a decaying total scattering density can be achieved if |Δ*ρ*_1_| < |Δ*ρ*_2_|, and the moduli of the scattering density contrasts increase with the distance *r* from the fiber axis (Figure 7). In principle, this radially decaying, periodic scattering density variation over (parts of) the fiber cross-section is related to the behavior of the polymer melt along the temperature gradient in the fiber. For example, different (radial) cooling rates can lead to changes in the free volume between adjacent polymer chains, which is additionally influenced by the radial extrusion velocity profile of the polymer melt. Furthermore, internal stress, which is likely to occur in the outer rim of the fiber cross section may—in combination with the velocity profile—possibly induce ring segments of different density separated by nanoscopic cracks. Albeit the origin of such radially decaying, periodic scattering density contrast variations over (parts of) the fiber cross-section—which is clearly indicated by the equatorial real-space correlation peak—is far from being understood, the described model appears to be self-consistent, and can at least be used to correctly interpret the structural information contained in the γ_equ_(*r*) in terms of the model function:

(9) $$I_{\mathrm{eq}}\left(q_{r}\right)=S_{0}{\left\{2\pi \sum_{k=1}^{2N}f\left(k,N,N_{R0}\right)\Delta \rho_{k}\left[\frac{J_{1}\left(q_{r}R_{k}\right)}{q_{r}R_{k}}R_{k}^{2}-\frac{J_{1}\left(q_{r}R_{k-1}\right)}{q_{r}R_{k-1}}R_{k-1}^{2}\right]\right\}}^{2},$$

where *S*_0_ is a scaling factor, *J*_1_ is the first order Bessel function, *N* is the number of repeat units, *R*_0_ is the radius of the fiber core with constant scattering density (i.e., Δ*ρ*_0_ = 0), *R*_1_ = *R*_0_ + Δ_1_, *R*_2_ = *R*_1_ + Δ_2_, *R*_k_ = *R*_k−1_ + Δ_1_ if *k* = 2*n* − 1, and *R*_k_ = *R*_k−1_ + Δ_2_ if *k* = 2*n*. The scattering contrast Δ*ρ*_k_ equals Δ*ρ*_1_ if *R*_k_ − *R*_k−1_ = Δ_1_, and Δ*ρ*_k_ = Δ*ρ*_2_ if *R*_k_ − *R*_k−1_ = Δ_2_.

The function *f*(*k*,*N*,*N*_R0_) increases the moduli of the scattering contrasts Δ*ρ*_k_ with increasing *k*, i.e.:

(10) $$f\left(k,N,N_{R0}\right)=\frac{k+2N_{R0}}{2N_{\mathrm{tot}}}-\frac{N_{R0}}{N_{\mathrm{tot}}},$$

where *N*_R0_ is the number of repeat units that fit into the radius *R*_0_, i.e., *N*_R0_ = *R*_0_/(Δ_1_ + Δ_2_), and *N*_tot_ is the total number of repeat units for a fiber with radius *R*_f_, i.e., *N*_tot_ = *R*_f_/(Δ_1_ + Δ_2_). 

Equation (9) is a very simple approach to the above described model and is in its current form not applicable to fit the observed equatorial SAXS data. The development of a suitable model—similar to the one known for stacked platelets [34]—requires much more effort in order to separate the single repeat unit scattering (i.e., the “single-particle” scattering) from the structure factor that describes the scattering contributions arising from the interference of different repeat units, which is necessary to introduce corrections such as, e.g., size-distribution functions for the repeat units, and uncertainty factors for the inter-particle scattering into the model scattering function. Nevertheless, the model curves demonstrate that the information content in the equatorial Patterson function is basically the same for a GI-POF (where the density variation starts at *R*_0_ = 0 nm) or a multi-step index (MSI)-POF (where the density variation starts for the example structure at *R*_0_ = 3800 nm, see Figure 7).

The *D*_MAX_ value corresponds to the doubled width of the smaller ring-segment, i.e., *D*_MAX_ = 2Δ_min_ = 2**Δ**_2_ and the position of the correlation peak *D*_tot_ is given by:

(11) $$D_{\mathrm{tot}}=\sqrt{2\left(\Delta_{1}^{2}+\Delta_{2}^{2}\right)}$$

The comparison of the values *R*_tot_ = *D*_tot_/2 with the results for the cylinder radii *R*_c_ obtained from the Guinier analysis (see Table 2) agree almost perfectly for all four POF samples, which demonstrates, once again, that the results are consistent, and, more important, that the above described equatorial structure model is a simple but reasonable approach to the real nanostructure of the POFs over the fiber cross-section.

**Figure 8:**
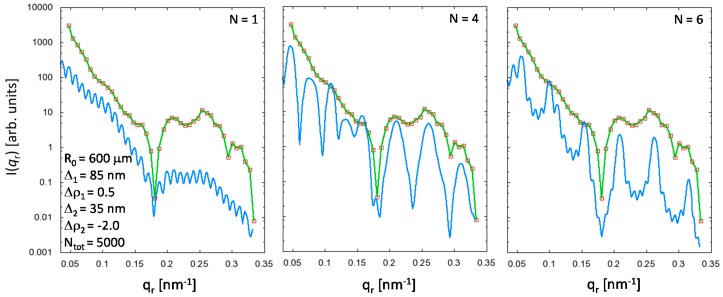
Simulated SAXS intensities (blue solid lines) in comparison with the observed data (symbols and green solid lines) for the sample POF1 using the structure parameters displayed in the inset on the left for different numbers *N* of repeat units. For better visibility, high-frequency oscillations in the model SAXS curves were suppressed by a seven-point moving average. The model curve with *N* = 6 repeat units reproduces the positions of the maxima and minima in the measured data quite well. A POF1 sample consisting of only one single repeat unit (*N* = 1) is not very likely.

A simple simulation using Equation (9), where the parameters **Δ**_1_ = 85 nm and **Δ**_2_ = 35 nm were manually found by variation of the respective values derived from the equatorial Patterson function within their uncertainties, was carried out for the sample POF1 which displays the most pronounced features in the equatorial scattering curve (see Figure 8). Although the model scattering intensities can of course not even rudimentarily describe the measured data, it can yet be shown that the maxima and minima in the observed intensities are correctly reproduced and that the sample is a multi-step index fiber rather than a single-step or gradient-index POF (which is presumably valid for all four investigated PMMA POF samples). 

The upturn of the measured SAXS curve at very small *q*-values in comparison to the model curve (Figure 8) may either be indicative of additional scattering contributions arising from micro cracks or gas-inclusions, which also need to be taken into account for a proper data modeling, and/or can be attributed to an incorrectly chosen fiber core radius *R*_0_ (see the SAXS curves in Figure 7). To access information on such large-scale structure features for an improved interpretation of the results (for both equatorial and meridional scattering intensities) requires an extension of the experiments into the ultra-small-angle X-ray scattering (USAXS) regime, i.e., *q* ≤ 10^−2^·nm^−1^, making use of appropriate techniques such as, e.g., light scattering and/or USAXS-cameras (Bonse-Hart) [35,36]. Regardless of these limitations, the comparison between model curve and observed data clearly demonstrates that the parameters derived from Guinier and real-space analysis are related to the PMMA nanostructure rather than to scattering contributions originating from cracks or voids. This indication can in principle be easily confirmed if the structure parameters show systematic dependencies on the processing conditions. Moreover, strong correlations between process and structure parameters are a clear sign that the polymer structure, and, in particular, the radial density profile, can be modified by changes in the fiber production process.

As already mentioned above, single process parameters were not systematically varied in this study. However, since the overall characteristics for the production of the four fiber samples are different, at least integral structure—process relationships can be obtained, which require the definition of an overall process parameter that (qualitatively) reflects the interdependencies of the complete set of production factors with respect to their relative impact on the formation of the fiber structure.

The variation of the spin-draw ratio λ_SD_ has a strong influence on the structure of a fiber material that is rapidly cooled down, and almost no influence on the structure of a fiber in the molten state. Consequently, it appears reasonable to weight the spin-draw ratio λ_SD_ with the temperature of the water-bath *T*_water_. This argument also holds for the mechanical draw ratio λ_D_, which can be weighted with the drawing temperature *T*_D_. The effect of the applied mechanical draw ratio λ_D_ on the fiber structure also depends on the history of the as-spun material, since the relative impact of the draw ratio on the fiber nanostructure is higher if the fiber is spun at a moderate spin-draw ratio, and decreases with increasing spin-draw ratio.

**Figure 9:**
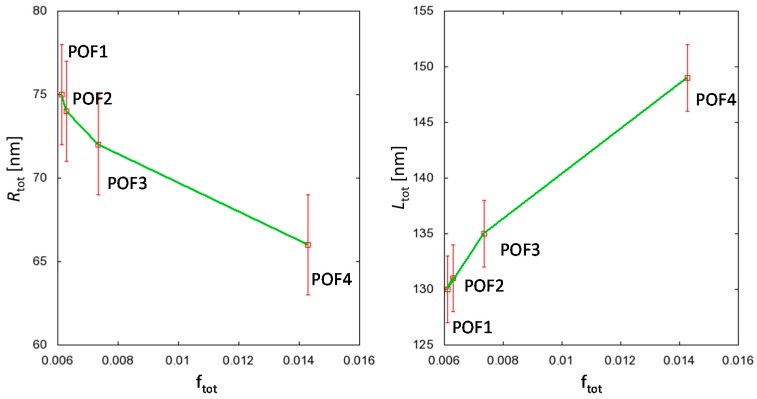
Dependence of the integral equatorial and meridional parameters *R*_tot_ (**Left**) and *L*_tot_ (**Right**) on the overall process-parameter *f*_tot_. Although the major differences in the structure parameters are induced by the mechanical draw ratio, the impact of small changes in the spin-draw ratio between POF1 and POF2 is also noticeable.

Thus, an approximate overall process parameter *f*_tot_ can be created which is given by:

(12) $$f_{\mathrm{tot}}=\frac{\lambda_{D}}{\lambda_{\mathrm{SD}}}\frac{\left(T_{\mathrm{water}}-T_{0}\right)}{\left(T_{D}-T_{0}\right)},$$

where *T*_0_ corresponds to ambient temperature. The overall factor *f*_tot_ defined by Equation (12) has to be considered as a very simple approximation to the real dependencies of the single process parameters. Though the major changes in the integral structure parameters are due to the mechanical draw ratio (which is in fact of minor importance for the fabrication of POFs, see Table 1), Figure 9 reveals that *R*_tot_ and *L*_tot_ depend (even for the comparatively small changes in the process parameters between POF1 and POF2) systematically on *f*_tot_, which proves that the equatorial (scattering-) density, as well as the meridional length distribution of the straight PMMA chain segments in the fiber, can be manipulated by selection of the processing conditions. 

## 5. Conclusions

Four PMMA-POFs produced with different sets of process parameters were investigated with small-angle X-ray scattering (SAXS). The SAXS intensity distributions were analyzed by a Guinier approach and the computation of the distance distribution functions in order to extract information on the PMMA fiber nanostructure in the equatorial plane and along the fiber axis. The occurrence of real-space correlation peaks in both equatorial and meridional distance distribution functions suggest periodic variations of the scattering density along and perpendicular to the fiber axis in the amorphous POFs, and the resulting structure parameters can be assigned to scattering density variations over the fiber cross-section and the occurrence of straight PMMA chain segments along the fiber axis. The integral structure parameters are clearly correlated to—and, therefore, controllable by—changes in the processing conditions. A simple model that describes the radially decaying periodic density variation is found to reproduce the essential structural features of the equatorial scattering curves. Though the mechanism that induces these radially decaying periodic changes in the equatorial scattering density contrast is yet to be investigated in detail, the origin of the fiber cross-sectional refractive-index profiles is definitely a radial density gradient. Despite the fact that the explored range of processing conditions is, by far, too small to derive correlations that are of real significance, this very first SAXS study on the response of the PMMA fiber structure to variations of the processing conditions can be considered successful, since it shows that SAXS experiments can indeed contribute to the determination of structure–property process relationships, which may be exploited to finally fabricate POFs with well-defined refractive-index profiles that can be designed by the choice of appropriate processing conditions.

## Figures and Tables

**Table 1:** Fiber processing conditions for the four POF samples selected for the SAXS analysis.

Sample No.	Nozzle diameter (mm)	*v*_w_ (m/min)	λ_SD_ (= *v*_w_/*v*_e_)	*T*_water_ (°C)	λ_D_ (= *l*_D_/*l*_0_)	*T*_D_ (°C)
1	0.8	45	18.85	25	3	150
2	2.0	7	18.32	25	3	150
3	2.0	20	52.36	35	5	150
4	0.8	45	18.85	25	7	150

**Table 2:** Resulting equatorial and meridional characteristic dimensions of the investigated POF samples obtained from distance distribution function (γ) and Guinier approximation (G).

Sample No.	Δ_min_ (γ) (nm)	*R*_tot_ (γ) (nm)	*L*_SC_ (γ) (nm)	*L*_tot_ (γ) (nm)	*R*_c_ (G) (nm)	*L*_SC_ (G) (nm)	*L*_EC_ (G) (nm)
1	33 (3)	75 (3)	50 (2)	130 (2)	75 (4)	45 (2)	80 (4)
2	32 (3)	74 (3)	51 (2)	131 (2)	74 (4)	44 (2)	79 (4)
3	32 (3)	72 (3)	50 (2)	135 (2)	68 (3)	47 (2)	77 (4)
4	31 (3)	66 (3)	49 (2)	149 (2)	63 (3)	46 (2)	84 (4)

## References

[1] Bunge C.-A., Beckers M., Gries T. (2016). Polymer Optical Fibres: Fibre Types, Materials, Fabrication, Characterisation and Applications.

[2] Asai M., Inuzuka Y., Koike K., Takahashi S., Koike Y. (2011). High-Bandwidth Graded-Index Plastic Optical Fiber with Low-Attenuation, High-Bending Ability, and High-Thermal Stability for Home-Networks. J. Lightwave Technol..

[3] Gloge D., Marcatili E. (1973). Multimode theory of graded-core fibers. Bell Syst. Tech. J..

[4] Freund R.E., Bunge C.-A., Ledentsov N.N., Molin D., Caspar C. (2010). High-speed transmission in multimode fibers. J. Lightwave Technol..

[5] Beckers M., Schlüter T., Vad T., Gries T., Bunge C.-A. (2015). An overview on fabrication methods for polymer optical fibers. Polym. Int..

[6] Beckers M., Vad T., Steinmann W., Holt N., Schlüter T., Gries T., Bunge C.-A. Herstellung von Gradienten-Index-Polymer-Fasern mit reproduzierbaren (optischen) Eigenschaften im Industriemaßstab: Ein Schmelzspinn-Verfahren. Proceedings of the 30. Fachgruppentreffen der ITG-FG 5.4.1 “Optische Polymerfasern”.

[7] Bunge C.-A., Beckers M., Gries T., Bremer T., Roth B. Dopant-free Fabrication Process for Graded- Index Polymer Optical Fiber Solely Based on Temperature Treatment. Proceedings of the IEEE 17th International Conference on Transparent Optical Networks (ICTON).

[8] Shakher C., Nirala A.K. (1999). A review on refractive index and temperature profile measurements using laser-based interferometric techniques. Opt. Lasers Eng..

[9] Baeumer S. (2005). Handbook of Plastic Optics.

[10] Pendke P., Das K. (2015). Variation of Refractive Index PMMA with temperature and different doping % of TiO_2_. Int. J. Sci. Res. Manag..

[11] Boehm J., Haußelt J., Henzi P., Litfin K., Hanemann T. (2004). Tuning the refractive index of polymers for polymer waveguides using nanoscaled ceramics or organic dyes. Adv. Eng. Mater..

[12] Blyler L.L., Koeppen C.S., Bair H.E. GI-POFs in telecommunications: Opportunity and reliability. Proceedings of the POF Asia-Pacific Forum.

[13] Rysselberghe P.V. (1932). Remarks concerning the Clausius–Mossotti Law. J. Phys. Chem..

[14] Feigin L.A., Svergun D.I. (1987). Structure Analysis by Small-Angle X-ray and Neutron Scattering.

[15] Zubia J., Arrue J. (2001). Plastic optical fibers: An introduction to their technological processes and applications. Opt. Fiber Technol..

[16] Bunge C.-A., Beckers M., Gries T. Simple and Adjustable Fabrication Process for Graded-Index Polymer Optical Fibers with Tailored Properties for Sensing. Proceedings of the IEEE Sensors.

[17] Bunge C.-A., Kruglov R., Poisel H. (2006). Rayleigh and Mie Scattering in Polymer Optical Fibers. J. Lightwave Technol..

[18] Goode W.E., Owens F.H., Fellmann R.P., Snyder W.H., Moore J.E. (1960). Crystalline Acrylic Polymers. I. Stereospecific Anionic Polymerization of Methyl Methacrylate. J. Polym. Sci..

[19] Nishioka A., Watanabe H., Abe K., Sono Y. (1960). Grignard Reagent-Catalyzed Polymerization of Methyl Methacrylate. J. Polym. Sci..

[20] Pauw B.R., Vigild M.E., Mortensen K., Andreasen J.W., Klop E.A. (2010). Analysing the nanoporous structure of aramid fibres. J. Appl. Cryst..

[21] Haubold H.-G., Grünhagen K., Wagner M., Jungbluth H., Heer H., Pfeil A., Rongen H., Brandenberg G., Moeller R., Matzerath J. (1989). Jusifa—A new user-dedicated ASAXS beamline for materials science. Rev. Sci. Instrum..

[22] Kentzinger E., Krutyeva M., Rücker U. (2016). GALAXI: Gallium anode low-angle X-ray instrument. J. Large-Scale Res. Facil..

[23] Stribeck N. (2007). X-ray Scattering of Soft Matter.

[24] Laaziri K., Kycia S., Roorda S., Chicoine M., Robertson J.L., Wang J., Moss S.C. (1999). High-energy X-ray diffraction study of pure amorphous silicon. Phys. Rev. B.

[25] Fukasawa T., Sato T. (2011). Versatile application of indirect Fourier transformation to structure factor analysis: From X-ray diffraction of molecular liquids to small angle scattering of protein solutions. Phys. Chem. Chem. Phys..

[26] Press W.H., Teukolsky S.A., Vetterling W.T., Flannery B.P. (1992). Numerical Recipes in Fortran.

[27] Litvinov V.M., Xu J., Melian C., Demco D.E., Möller M., Simmelink J. (2011). Morphology, Chain Dynamics, and Domain Sizes in Highly Drawn Gel-Spun Ultrahigh Molecular Weight Polyethylene Fibers at the Final Stages of Drawing by SAXS, WAXS, and ^1^H Solid-State NMR. Macromolecules.

[28] Heeley E.L., Hughes D.J., Crabb E., Kershaw M., Shebanova O., Leung S., Mayoral B., McNally T. (2016). Structure evolution in poly(ethylene terephthalate) (PET) Multiwalled carbon nanotube (MWCNT) composite films during in-situ uniaxial deformation. Polymer.

[29] Hosemann R., Bagchi S.N. (1962). Direct Analysis of Diffraction by Matter.

[30] Strobl G.R., Schneider M. (1980). Direct Evaluation of the Electron Density Correlation Function of Partially Crystalline Polymers. J. Polym. Sci. Polym. Phys..

[31] Balzano L., Kukalyekar N., Rastogi S., Peters G.W.M., Chadwick J.C. (2008). Crystallization and dissolution of flow-induced precursors. Phys. Rev. Lett..

[32] Graham R.S., Olmsted P.D. (2010). Kinetic Monte Carlo simulations of flow-induced nucleation in polymer melts. Faraday Discuss..

[33] Hsu H.P., Paul W., Binder K. (2012). Scattering function of semiflexible polymer chains under good solvent conditions. J. Chem. Phys..

[34] Kratky O., Porod G. (1949). Diffuse Small-Angle Scattering of X-rays in Colloid Systems. J. Colloid Sci..

[35] Bonse U., Hart M. (1965). An X-ray Interferometer. Appl. Phys. Lett..

[36] Bonse U., Hart M. (1966). Small Angle X-ray Scattering by Spherical Particles of Polystyrene and Polyvinyltoluene. Z. Phys..

